# The vertebrate retina: a window into the evolution of computation in the brain

**DOI:** 10.1016/j.cobeha.2024.101391

**Published:** 2024-06

**Authors:** Tom Baden

**Affiliations:** Center for Sensory Neuroscience and Computation, School of Life Sciences, University of Sussex, Brighton BN19QG, UK

## Abstract

Animal brains are probably the most complex computational machines on our planet, and like everything in biology, they are the product of evolution. Advances in developmental and palaeobiology have been expanding our general understanding of how nervous systems can change at a molecular and structural level. However, how these changes translate into altered function — that is, into ‘computation’ — remains comparatively sparsely explored. What, concretely, does it mean for neuronal computation when neurons change their morphology and connectivity, when new neurons appear or old ones disappear, or when transmitter systems are slowly modified over many generations? And how does evolution use these many possible knobs and dials to constantly tune computation to give rise to the amazing diversity in animal behaviours we see today? Addressing these major gaps of understanding benefits from choosing a suitable model system. Here, I present the vertebrate retina as one perhaps unusually promising candidate.

The retina is ancient and displays highly conserved core organisational principles across the entire vertebrate lineage, alongside a myriad of adjustments across extant species that were shaped by the history of their visual ecology. Moreover, the computational logic of the retina is readily interrogated experimentally, and our existing understanding of retinal circuits in a handful of species can serve as an anchor when exploring the visual circuit adaptations across the entire vertebrate tree of life, from fish deep in the aphotic zone of the oceans to eagles soaring high up in the sky.

## Highlights

• Vertebrate retinal circuits including many specific neuron types are highly conserved.
• Different species exhibit key circuit adaptations for their specific visual ecology.
• Retinal computations represent a complexity sweet spot and are readily interrogated.

**Current Opinion in Behavioral Sciences** 2024, **57**:101391

This review comes from a themed issue on **Brain Evolution: from cell types to behavior**

Edited by **Maria Antonietta Tosches** and **Lucia Prieto-Godino**

For complete overview of the section, please refer to the article collection, “Brain Evolution: from cell types to behavior (2023)”

https://doi.org/10.1016/j.cobeha.2024.101391

2352–1546/© 2024 The Author(s). Published by Elsevier Ltd.This is an open access article under the CC BY license (http://creativecommons.org/licenses/by/4.0/).

## Introduction

Understanding computation in the nervous system is hard, but understanding its evolution is arguably harder 1••, 2. Evolution is a process of change, and therefore requires an understanding of both ‘the before’ and ‘the after’. However, ‘the before’ is no more. The usually next best option, therefore, is to study two or more different states of ‘the after’, and then use their results to infer what might have existed in the past. This issue compounds with the fact that evolution is slow when contrasted against the pace of scientific endeavour or our own lifespan. This means that the possibility for experimental evolution [3], where we might causally interfere with the evolutionary process, remains largely restricted to species with very short generation times. And even where this is possible, it is difficult to know how whatever new solution evolves relates to what did or would have happened in the past or in different environmental conditions.

**Figure 1:**
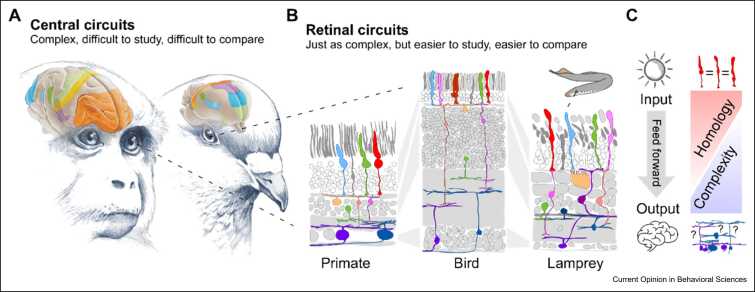
The brain, the eye, and the retinal circuit organisation. **(a)** The neural computations of central circuits are usually difficult to study and link across large phylogenetic distances. **(b)** By comparison, deep circuit conservation across all vertebrate retinas helps link circuit elements across large evolutionary distances. **(c)** Retinal circuits are feedforward across the layers, from the outer to the inner retina and finally, the spiking output of retinal ganglion cells to the brain [8]. Retinal circuit elements are also highly conserved, with neuron types falling along a homology gradient that peaks with photoreceptors and horizontal cells in the outer retina and gradually falls via bipolar cells to the amacrine cells and ganglion cells [12]. This homology gradient antialigns with a complexity gradient, both in terms of neuronal diversity and in terms of the visual signals that neurons at different stages of retinal processing encode. Photoreceptors carry comparatively simple and broad signals, while visual features encoded by ganglion cells can be notably more specific.

The bottom line of these basic considerations is that for understanding the evolution of computation in the nervous system, we should give ourselves every possible advantage. Most important, perhaps, is choosing the ‘right’ model system. In this essay, I will present the retina 4, 5, 6, 7 as a promising discovery platform for studying the evolution of computation in the vertebrate nervous system (Figure 1a–c). I will argue that this is because the vertebrate retina is exquisitely well suited for studying neural computation in the first place 8, 9, 10, while its ancient origin 6, 11, deep circuit homologies across the entire vertebrate tree of life [12], and intimate link to visuo-behavioural ecology 4, 13, 14, 15, 16, 17 mean that we can build upon our existing understanding of retinal computations in a few species [18] to grow our understanding of how neural computation can evolve in general.

**(a)** Adapted from Ref. [19]. **(b)** Adapted from Refs. 4, 18.

### Accessibility

Meaningfully understanding neural computation usually requires digging into the activity patterns of small numbers of neurons and their compartments [20], and this benefits from restricting efforts to a small part of the nervous system. Working on the sensory periphery has the advantage that this allows excellent control over the input while working on the late stages of motor systems has the advantage that we might achieve an accurate readout of the output. Working on anything in between these two extremes, however, requires either measuring or inferring both the input and the output and usually, this is simultaneously more difficult and less accurate.

Therefore, while ‘half’ of the problem of knowing both the input and the output is relatively easily solved for both sensory and motor systems, the crux is the respective other half. On balance, sensory systems have an additional leg-up in this regard; many sensory systems can, to some approximation, continue to function normally even if unplugged from the rest of the nervous system. In the case of the vertebrate eye, this is particularly straightforward; it typically means isolating the eye and/or retina and recording light-driven spike patterns from retinal ganglion cells that form the optic nerve [8].

### A complexity sweet spot

Beyond accessibility, a second key consideration is complexity. The ideal system should be ‘complex enough’ to be interesting and representative of neural computation elsewhere in the brain, but at the same time it needs to be ‘simple enough’ such that it is possible to gain useful insights in the first place. Here, the vertebrate retina, which develops from and is part of the central nervous system [6], arguably represents a complexity sweet spot amongst the senses; like the cortex or many other key brain structures, it is a fundamentally layered structure, and it is the only sensory tissue that houses two synaptic layers right in the periphery [21], before digitisation for transmission to the brain. This architecture allows the retina to preprocess the visual information ‘twice’ before digitising the signal for transmission to the brain. Correspondingly, sensory signals travelling down the axons of retinal ganglion cells that form the optic nerve are preprocessed to a degree that would be difficult to achieve in other sensory systems. Moreover, this is accomplished using a structural substrate that is at least conceptually in line with the layered organisation of much of the rest of the brain [22]. However, with one additional advantage: unlike in most other parts of the brain, signal propagation across retinal layers is almost entirely feedforward 8, 10. This means that computations can be systematically interrogated at multiple strictly subsequent processing stages — for example, at the level of the outer retina, then the inner retina, and finally, the spiking signal on the optic nerve [10]. The power of the ability to watch the visual signal change as it traverses the retinal circuit is difficult to overstate. Not only does it mean that we can choose to work at different levels of computational complexity all within the same tissue simply by picking the ‘right’ processing layer, but it also means that we can causally interfere with individual circuit elements while reading out their immediate downstream consequences.

Another distinguishing feature of the eye is that spatial information comes ‘for free’. This is because the retina is essentially a flat piece of tissue that is made from repeating units [23], where eye optics ensure that different parts of the physical image fall onto different parts of this network. Where other sensory systems are forced to infer information about space — for example, using interaural signal differences in the case of ears — in vision, space is fundamentally inbuilt and can be immediately used to extract higher-order compound features such as motion [24]. Retinal computations that ultimately enable complex feature representation such as directional motion selectivity are also critically dependent on the input from laterally connecting regulatory networks right within the eye 25, 26, 27, 28, 29, 30 — horizontal cells in the outer retina and amacrine cells within the inner retina. Accordingly, where feedback signals are sparse or absent across the layers, they abound within. In fact, from a perspective of neuron-type diversity, the vertebrate retina is arguably just as complex as any other part of the brain. For example, in mouse, the retina houses in the order of 140 types of neurons 12••, 31, 32••, 33 — compared with ∼90 neuron types that are thought to make up its primary visual cortex [34].

In many ways, both poetic and concrete, the retina therefore is a window to the brain. It is part of the brain and arguably just as complex, while at the same time being substantially more experimentally accessible and organised in a manner conducive to detailed functional interrogation. It should therefore come as no surprise that the retina is probably the most well-understood complex neuronal network of the vertebrate nervous system [35]. For studying the evolution of computation, we can build on this knowledge by choosing circuits or computations that are already well understood in one species, to assess if and how these are different in another species.

### The retina is ancient

All extant vertebrates, from lampreys to eagles, share a common fundamental blueprint of retinal organisation 4, 6, 18 (Figure 1b,c). Five classes of neurons (photoreceptors, horizontal cells, bipolar cells, amacrine cells, and ganglion cells) are arranged into three nuclear layers that flank two synaptic layers [8]. Even substructure within these neuronal classes is essentially universal. For example, transcriptomic signatures for On versus Off groups of bipolar cells, or of Gamma-Aminobutyric Acid versus glycinergic amacrine cells, are always present [12] (see also Refs. 36, 37, 38, 39••). Next, within the individual types of neurons that make up each class, homology varies across the levels of retinal organisation. Photoreceptor homology is essentially perfect — to the point where we can note with confidence that the ‘red cones’ (expressing long wavelength sensitive [LWS] opsin) of lampreys are homologous to the red cones in our own eyes 7, 17, 40. On the other end, the individual types of ganglion cells are less obviously conserved over this span. Nevertheless, some ganglion cell types can be linked across substantial evolutionary distances. For example, all vertebrates studied to date retain closely related intrinsically photosensitive retinal ganglion cells [12]. Together, this means that the retina offers a systematic homology gradient 12••, 39••, 41, 42, 43 that antialigns with its complexity gradient 8, 9, 13, 29, 32••, 33, 35 (i.e. photoreceptors plus horizontal cells < bipolar cells < amacrine cells plus ganglion cells), and we can exploit these gradients to survey the evolution of computation at different degrees of complexity and over short or long phylogenetic distances, including to the very origin of complex vertebrate life in the Cambrian shallows, some 550 million years ago.

### Strong link to phylogeny and behavioural ecology

Next, when studying evolution, we do not only wish to understand the what and the how but also the why. In the evolution of vision, this primarily links to two basic considerations: visuo-behavioural ecology and phylogeny. Both can be measured or at least inferred with a good degree of accuracy, and in many cases, this link can be quantitative 13, 15, 44, 45, 46, 47, 48, 49, 50. This means that we can build a reasonably coherent picture of the visuo-ecological pressures that ultimately led to a specific type of retinal design (e.g. Refs. 17, 40, 51, 52, 53, 54, 55••).

**Figure 2:**
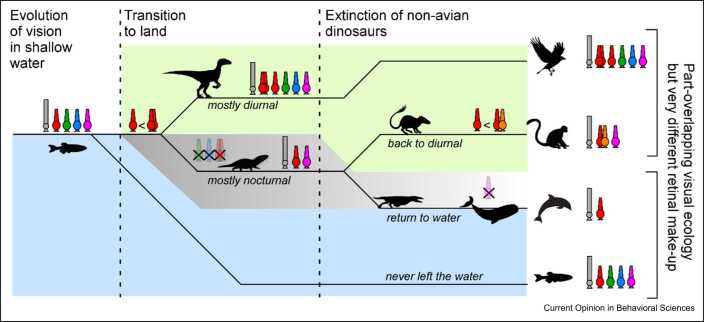
Retinal designs have been shaped by the history of an animal’s visual ecology. For example, some birds and primates, or some cetaceans and fish, share a common habitat and visual ecology. However, because of their distinct evolutionary histories, their retinas are built very differently. Vision evolved in the water for some 150 million years before the first vertebrates emerged on land, which probably came in hand with the emergence of a new pair of photoreceptors called the ‘double cone’ (expressing LWS), potentially to support ‘fast’ vision 51, 63. Soon after came the split of the early amniote lineage that would ultimately give rise to birds and reptiles on one branch and mammals on the other. Reptiles dominated diurnal niches, while early mammals were mostly nocturnal. During this time, the lineage that would give rise to modern eutherian mammals first lost the green cone (RH2), then blue (SWS2), and finally also the double cone (LWS). Upon the extinction of the dinosaurs, many mammals returned to a diurnal lifestyle, and some of them eventually gave rise to primates and ultimately humans. Very recently, primates duplicated their ancestral red cones (LWS) to evolve a relatively unusual form of *de novo* trichromacy [64]. By contrast, other mammals returned to the water, became very large, and lost the ancestral UV cone (SWS1). Some of these became small again to ultimately give rise to dolphins. All the while, fish never left the water, and many retain the complete ancestral photoreceptor complement. RH2, Rhodopsin-2; SWS, Short Wavelength Selective.

To take one simple example: unlike most fish, dolphins are colour blind [56], and we have a good idea why this should be the case (Figure 2). Vertebrate colour vision is usually based on the comparison of at least two spectrally distinct types of cones, and ancestrally, vertebrates have four: red, green, blue, and UV (LWS, Rhodopsin-2, Short Wavelength Selective 2, and Short Wavelength Selective 1, respectively) [17]. Many fish, including some that overlap in their visual ecology with that of dolphins, retain this ancestral tetrachromatic colour vision [57]. However, dolphins are descendants of terrestrial mammals, which in turn reduced their cone complement down to two [58] during the age of the dinosaurs. From here, probably still cone-dichromat mammals re-entered the water, and over time, they became enormous [59]. With an increase in body size comes an increase in visual interaction distance. For vision to be useful for a large whale, it needs to inform about visual structure at least a few metres away. However, underwater light becomes increasingly monochromatic with distance [60], and in the open ocean beyond a few metres distance, what little colour information there might have been to begin with is essentially gone [61]. Moreover, large whales are capable of diving to great depths, where light sparsely penetrates. Likely as a direct consequence of one or several of these factors, early whales lost another cone type and the ability to see colour along with it [62]. This would then also mean that any postsynaptic circuits that previously existed to contrast cone types would have been either lost or co-opted for other purposes. Later, the lineage that ultimately would lead to modern dolphins became smaller again and adopted a lifestyle closer to the surface where colour vision might again be useful. However, the second cone was long lost, and no compensatory mechanisms are known to have since re-appeared.

### Photoreceptors as a natural point of entry

**Figure 3:**
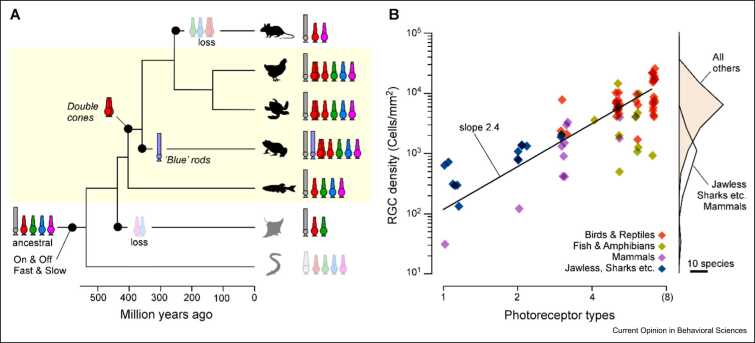
Phylogeny, photoreceptor types, and retinal complexity. **(a)** Phylogenetic tree of major vertebrate lineages with key events in the evolution of photoreceptor types and features indicated. **(b)** The density of vertebrates’ retinal ganglion cells scales with the number of photoreceptor types present. By and large, vertebrates can be divided into two retinal complexity groups: mammals, elasmobranchs (sharks, rays, and skates), and jawless species typically have low photoreceptor type diversity and low-density retinas, while many fish, amphibians, reptiles, and birds have larger numbers of photoreceptor types and high-density retinas.

While dolphins are therefore cone monochromats, they are but one example in an ocean of possibilities with regards to modifying the retina’s photoreceptor complement 17, 40, 65. Many species have lost different subsets of ancestral cones and/or rods, while others have expanded upon them in instructive ways ( Figures 2 and 3a). For example, early tetrapods evolved an extra pair of red cones called the double cone 40, 63, 66•• (which was later again lost in eutherian mammals). The two members of this double cone wire into the rest of the retina independent of each other, and both also wire independent of the ancestral red single cones 66••, 67. Bird retinas are therefore driven by three types of red cones, which brings their full photoreceptor complement up to seven — more than twice that of mammals. What might be the consequences of driving a retina with seven independent inputs, as opposed to three as in the case of, for example, mice?

**(a)** Mod from Ref. [51]. **(b)** Based on Ref. [17].

A survey across comparative studies hints that a species’ photoreceptor-type diversity may be fundamental to how the rest of the retina is organised [17]. For example, diurnal birds typically comprise some 10-fold more inner retinal neurons per equivalent area compared with mammals or sharks 4, 18 (Figure 3b), and many of these ‘extra’ avian neurons are moreover multistratified across both the outer and inner retina [51]. By contrast, most mammalian retinal neurons are mono- or at most bistratified. Diurnal fish and amphibians usually sit in between these two extremes, both in terms of photoreceptor diversity and of anatomical complexity. Moreover, photoreceptor-type diversity also appears to scale with retinal complexity within more narrow phylogenetic groups. For example, unlike most diurnal birds, owls use fewer than seven photoreceptor types for their predominately nocturnal lifestyle [68], and correspondingly, they also feature notably fewer inner retinal neurons. Together, these observations hint that by and large, and independent of phylogeny, retinal anatomical complexity scales with the number of input channels (Figure 3a,b).

The same pattern also appears to be mirrored at the level of function. For example, mammals are generally thought to send a highly decorrelated signal to the brain 47, 49, 69, 70, 71, 72; by and large, On is segregated from Off, fast is segregated from slow, and ‘colour’ is segregated from ‘greyscale’. This strategy has the advantage that it is energy efficient, for example, because firing rates in individual ganglion cells can remain low. Birds, and to some extent also fish 30, 54, 73, appear to do the exact opposite. Most avian ganglion cells are On-Off, and the On and Off fractions in these cells encode different things [51]. The Off part is typically fast and spectrally simple, while the On fraction is slow and spectrally complex. In this way, birds effectively ‘multiplex’ temporal and spectral information through single axons — perhaps a necessary adaptation to keep the already large number of ganglion cells in check. In this way, birds are bandwidth efficient; however, for this code to work, the ganglion cells need to fire a lot of spikes. Perhaps this in turn is made possible by the generally lower per-spike energy demands in avian neurons compared with mammals [74]. Nevertheless, what these considerations might mean for specific inner retinal circuits, or indeed for how ‘the retinal code’ is built and ultimately used, remains largely unexplored. Here, the use of ancestral and derived cone types as a ‘way in’ to identify potentially related retinal circuits may prove to be instructive (Figure 3).

### Deep circuit homologies from the retina to the brain

Beyond photoreceptors at the input, and patterns of spikes in the population output, our understanding of a species’ current visual ecology and its history is also readily applied to inner retinal circuits. For example, the structural organisation of the midget and parasol systems that dominate our own visual experience 23••, 75 can be intimately linked to the history of our visuo-behavioural ecology. Like the aforementioned dolphins, we are the descendants of nocturnal mammals; however, we took a different path: our ancestors remained on land and adopted a diurnal lifestyle [58]. Our arboreal behavioural repertoire increasingly demanded an ability first for accurate grasping and later also for complex object manipulation. These demands meant that our eyes needed to become increasingly foveated. All the while, coupled with a gradual enlargement of the cortex, came a progressively expanding dependence on bilateral thalamic over the tried and tested contralateral collicular pathways, and this in turn probably required a different overall visual code — one that was increasingly general.

Today’s result of these types of pressures is a retina that is overwhelmingly dedicated to the production of two main types of information: spatial and temporal detail. These are represented by the midget and the parasol systems, respectively [48]. A good sense of colour vision was also increasingly useful for our recent ancestors, and to meet this need, the ancestral ‘red’ LWS-cone was duplicated, giving rise to a new ‘green’-LWS cone 40, 58, 64. This re-enabled trichromatic colour vision despite our own dichromatic ancestry. However, unlike in other species that share aspects of their visual ecology such as some birds, primate ‘red’ and ‘green’ cones are molecularly indistinguishable from each other beyond the opsin itself [42]. This meant that retinal circuits could not readily evolve to selectively read out each cone type in isolation. And even if they could, presumably the ‘original’ circuits that once enabled contrasting red versus green cones were long co-opted or lost. And yet, evolution will find a way. In our case, the probably already well-established midget system came to the rescue; because of small cone convergence, especially in the fovea, each midget ganglion cell is almost inevitably ‘red’ or ‘green’ biased 76, 77, and this bias can be readily learnt by the cortex during infancy [78]; unlike in fish, where the ‘colour-system’ is developmentally hardwired and thus works straight away, it takes human infants a few months to get it right. However, once learnt, human colour vision around the red–green axis is perfectly adequate [79], and perhaps superior to what we believe to be the spectral resolution achieved by some of the more ancestral solutions.

On the face of it, the heavy reliance on only two main systems (midgets and parasols) to do three main jobs (space, colour, and time) is a completely different visual strategy compared with that of fish, birds, or even of most non-primate mammals. And yet, as discussed in the following, both from an evolutionary perspective and from a point of view that seeks to understand how small-scale circuit level changes can lead to large-scale functional changes, it is not that far at all. Ultimately, the parasol and midget circuits probably reflect a very recent co-option of a small number of ancestral circuits that are common to all mammals, and perhaps even to fish. The origin, then, is ancient, and we can appreciate its path over evolutionary time by looking at the same circuit elements across multiple extant species that have variations of this system in the context of their own fascinating visual ecologies.

### Midget and parasol circuits over evolutionary time

**Figure 4:**
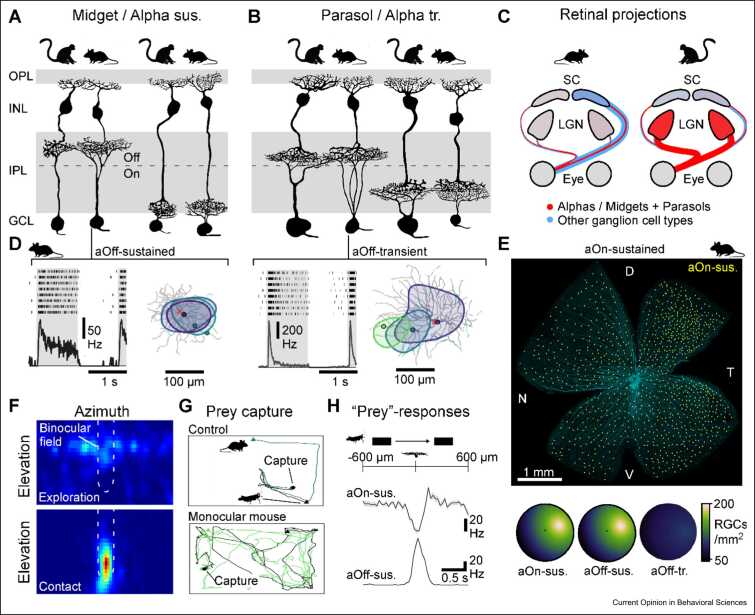
Evolution of the primate midget and parasol pathways. **(a,b)** Primate midget and parasol circuits are orthologs, respectively, of murine sustained and transient alpha ganglion cells and their presynaptic bipolar cells. **(c)** Retinal projections to the brain in mice and primates. **(d–h)** Murine-sustained alpha cells mirror key properties of primate midget circuits. Unlike transient alpha cells (right), sustained alphas display more sluggish light responses and electrotonically compact dendritic integration. Sustained but not transient alphas are enriched in the ventrotemporal retina to survey the upper-frontal horizon. A subset of these ventronasal-sustained alphas make ipsilateral projections that are probably key for visual prey capture of crickets. During hunting, mice align their heads to bring the target into their binocular zone **(f)**. Ablation of ipsilateral projection greatly deteriorates prey-capture performance **(g)**. On- and Off-sustained alpha cells readily respond to prey-like moving stimuli **(h)**.

Recent transcriptomic findings [12] suggest that the midget and parasol systems of our own eyes 23••, 80 are intimately linked with murine-sustained and transient alpha ganglion cells, respectively 81, 82 (Figure 4a–c). A link between the ‘fast and large’ parasols and the ‘fast and large’ transient alphas was long suspected; however, the discovery that the ‘slow and small’ midgets should find a counterpart with the ‘also quite fast and large’ sustained alphas was perhaps more of a surprise. And yet, based on what we now know, it makes a lot of sense!

**(a,b)** Mod from Ref. [12]. **(d)** Mod from Refs. 81, 88. **(e)** Mod from Ref. [92]. **(f–h)** Mod from Ref. [93].

Alpha cells were first described in cats [83], but they probably exist in all sighted mammals 12••, 82. For example, even hippos have them, and even here — like in the primate fovea — they seem to be enriched in both acute zones [84] (like other gigantic African mammals 85, 86, hippos have two acute zones, possibly an adaptation that reduces the need to move their heavy head). In the hippo, deeper insights into the specific subtypes of alphas or their functions remain outstanding, however, not so in mice. In fact, alongside direction-selective circuits [24], the mouse’s four types of alpha ganglion cells [81] and their presynaptic circuits 87, 88 represent some of the most intensely studied microcircuits in vertebrate neuroscience (e.g. Refs. 33, 89, 90, 91). These decades of work have uncovered a myriad of insights that we can now apply to explore the possible evolutionary links between the mouse’s sustained alphas and our own midgets. For example, unlike transient alpha cells, but like midgets, sustained alphas display electrotonically compact dendritic integration [88] (Figure 4d). Moreover, sustained but not transient alphas are enriched in the temporal retina to survey the frontal horizon, just above the nose [92] (Figure 4e). Indeed, a subset of these sustained alphas make ipsilateral projections that are probably key for visual prey capture of crickets [93], possibly the most ‘midget-like’ task in the mouse’s behavioural repertoire; during hunting, mice align their heads to bring the target into their binocular zone (Figure 4f), and ablation of ipsilateral ganglion cell projections greatly deteriorates prey-capture performance [93] (Figure 4g). Of the mouse’s ∼40 types of retinal ganglion cells 32••, 33, only a small subset project ipsilaterally, and these notably include all four types of alphas. In line, both On- and Off-sustained alpha cells readily respond to prey-like stimuli [93] (Figure 4h).

Beyond mammals, transcriptomic signatures of the two Off alphas (but not On) also exist in zebrafish and chicken 12••, 51, 67, 94, and perhaps even in lamprey [39], hinting that this subset of alphas long predates the emergence of mammals. However, in the absence of a clear genetic alpha marker in these nonmammalian species, functionally linking Off-alpha-like circuits over these larger phylogenetic distances remains difficult — though perhaps not impossible; in chicken and zebrafish, Off circuits are generally more suited for encoding temporal detail compared with On 30, 51, 73, 95. This functional signature has been taken to the extreme in chicken, where the encoding of fast temporal contrast appears to be the exclusive remit of the Off system [51]. Here, returning to the earlier argument on cones, studying putative alpha-like circuits in nonmammalian species might ultimately prove to be instructive in ways that could not otherwise emerge; mammalian eyes are overwhelmingly driven by ancestral red cones (LWS) and rods, and this drive naturally extends to all the alphas. However, this also means that from studying mammals alone, it is not possible to tell if alpha circuits are categorically red cone driven, or if ancestrally they might have integrated more broadly across available photoreceptors. Here, a quick look at zebrafish and chicken, who both retain the full four-cone ancestral photoreceptor complement 54, 67, hints that the red cone–dominated drive is in fact the ancestral state for alphas; fast Off circuits in these species are almost exclusively red cone driven 30, 51, 52, 60, 73, despite the easy availability of several other cone types. This tentatively suggests that there is a benefit of keeping these very ‘general’ alpha channels free from excessive cone pooling. In line, the mouse-sustained Off alpha is mainly driven by the UV-cone avoiding type-1 bipolar cell 12••, 96••, 97, and the sustained On alpha selectively avoids the processes of the UV-cone exclusive type-9 bipolar cell [97]. Perhaps, then, alpha cells are derived from ancient LWS-cone circuits that already helped our earliest aquatic ancestors navigate their newfound underwater worlds [17].

### Evolution of direction elective circuits

Beyond alphas, also the much-studied direction-selective circuits of rodent and rabbit retinas [98] appear to have been at least partially retained in our own eyes 99, 100. Direction-selective ganglion cells are also a feature of salamander [101] and fish retinas 102, 103, 104; however, if and how these link with mammalian direction-selective circuits remains unknown [105]. Similarly, determining if direction-selective circuits exist in even older lineages such as in sharks and lampreys, or if they persist in avian retinas — for example, to help control flight — will be important to address in the future. However, one thing is clear: signatures of the cholinergic amacrine cells [26] that centrally underpin the computation of motion direction in mammals [25] are found in all vertebrates (e.g. Refs. 39••, 106). In fact, these ‘starburst amacrine cells’ have been the subject of one of the perhaps most compelling studies on the evolution of computation in the nervous system to date; mice and rabbits have very different eye sizes, which means that the image of the same moving object will traverse the retinal surface at very different absolute velocities in each species. Correspondingly, visual motion circuits are tuned to very different absolute velocities in the two species’ eyes, and this adjustment is implemented via a nuanced spatial rearrangement of synaptic connectivity to starburst amacrine cells 53, 107. And yet, if these ancient cells have always been implicated in motion processing, or if rather they were co-opted much more recently for this purpose, remains to be established 105, 108.

## Funding

Funding was provided by the 10.13039/100004440Wellcome Trust (Investigator Award in Science 220277/Z20/Z), the 10.13039/501100000781European Research Council (ERC-StG ‘NeuroVisEco’ 677687), 10.13039/100014013UK Research and Innovation (10.13039/501100000268Biotechnology and Biological Sciences Research Council, BB/R014817/1 and BB/W013509/1), the 10.13039/501100000275Leverhulme Trust (PLP-2017-005 and RPG-2021-026 to TB), and the Lister Institute for Preventive Medicine (to TB). This research was funded in part by the Wellcome Trust (220277/Z20/Z). For the purpose of Open Access, the authors have applied a CC BY public copyright licence to any Author Accepted Manuscript version arising from this submission.

## Declaration of Competing Interest

None.

## Data Availability

No data were used for the research described in the article.
